# RETRACTION: The Effects of Evidence‐Based Nursing Interventions on Pressure Ulcers in Patients With Stroke: A Meta‐Analysis

**DOI:** 10.1111/iwj.70308

**Published:** 2025-03-06

## Abstract

**RETRACTION:** GaoM.‐M. , WangL.‐P. , ZhangL.‐L. , and LiY.‐Y. , “The Effects of Evidence‐Based Nursing Interventions on Pressure Ulcers in Patients With Stroke: A Meta‐Analysis,” International Wound Journal 20, no. 10 (2023): 4069–4076, 10.1111/iwj.14298.37438328 PMC10681431

The above article, published online on 12 July 2023, in Wiley Online Library (http://onlinelibrary.wiley.com/), has been retracted by agreement between the journal Editor in Chief, Professor Keith Harding; and John Wiley & Sons Ltd. Following an investigation by the publisher, all parties have concluded that this article was accepted solely on the basis of a compromised peer review process. The editors have therefore decided to retract the article. The authors did not respond to our notice regarding the retraction.

**RETRACTION:** M.‐M. Gao , L.‐P. Wang , L.‐L. Zhang , and Y.‐Y. Li , “The Effects of Evidence‐Based Nursing Interventions on Pressure Ulcers in Patients With Stroke: A Meta‐Analysis,” International Wound Journal 20, no. 10 (2023): 4069–4076, 10.1111/iwj.14298.37438328 PMC10681431

The above article, published online on 12 July 2023, in Wiley Online Library (http://onlinelibrary.wiley.com/), has been retracted by agreement between the journal Editor in Chief, Professor Keith Harding; and John Wiley & Sons Ltd. Following an investigation by the publisher, all parties have concluded that this article was accepted solely on the basis of a compromised peer review process. The editors have therefore decided to retract the article. The authors did not respond to our notice regarding the retraction.

